# 1-[3,5-Bis(trifluoro­meth­yl)phen­yl]-3-[(5-ethenyl-1-aza­bicyclo­[2.2.2]octan-2-yl)(6-methoxy­quinolin-4-yl)meth­yl]thio­urea–l-proline–methanol (1/1/1)^1^

**DOI:** 10.1107/S1600536809047072

**Published:** 2009-11-14

**Authors:** Savitha Muramulla, Hadi D. Arman, Cong-Gui Zhao, Edward R. T. Tiekink

**Affiliations:** a Department of Chemistry, The University of Texas at San Antonio, One UTSA Circle, San Antonio, Texas 78249-0698, USA; b Department of Chemistry, University of Malaya, 50603 Kuala Lumpur, Malaysia

## Abstract

In the methanol solvate of the title 1:1 cocrystal, C_29_H_28_F_6_N_4_OS·C_5_H_9_NO_2_·CH_4_O, the l-proline mol­ecule exists as a zwitterion. In the crystal, the disubstituted thio­urea, l-proline and methanol mol­ecules are linked by N—H⋯O and N—H⋯N hydrogen bonds, forming a two-dimensional array in the *ab* plane.

## Related literature

For background to pre-catalyst mol­ecules for the Michael addition of acetone to *trans*-β-nitro­styrene, see: Mandal & Zhao (2008 ▶). For a related structure, see: Muramulla *et al.* (2009 ▶). For discussion on the definition of a co-crystal, see: Zukerman-Schpector & Tiekink (2008 ▶). For the synthesis, see: Vakulya *et al.* (2005 ▶).
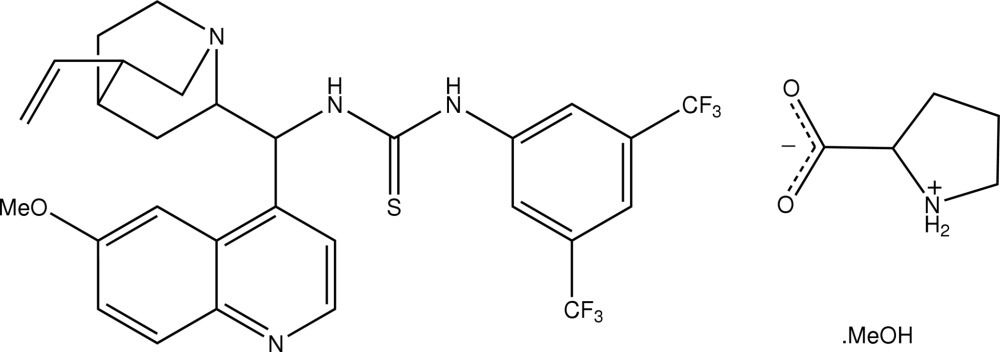


## Experimental

### 

#### Crystal data

C_29_H_28_F_6_N_4_OS·C_5_H_9_NO_2_·CH_4_O
*M* *_r_* = 741.79
Orthorhombic, 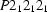

*a* = 11.597 (3) Å
*b* = 13.044 (4) Å
*c* = 23.907 (7) Å
*V* = 3616.4 (18) Å^3^
*Z* = 4
Mo *K*α radiation
μ = 0.17 mm^−1^
*T* = 98 K
0.28 × 0.25 × 0.05 mm

#### Data collection

Rigaku AFC12K/SATURN724 diffractometer
Absorption correction: multi-scan (*ABSCOR*; Higashi, 1995 ▶) *T*_min_ = 0.722, *T*_max_ = 1.000
26093 measured reflections
8250 independent reflections
7519 reflections with *I* > 2σ(*I*)
*R*_int_ = 0.059

#### Refinement

*R*[*F* ^2^ > 2σ(*F* ^2^)] = 0.060
*wR*(*F* ^2^) = 0.149
*S* = 1.06
8250 reflections
471 parameters
3 restraints
H-atom parameters constrained
Δρ_max_ = 0.44 e Å^−3^
Δρ_min_ = −0.27 e Å^−3^
Absolute structure: Flack (1983 ▶), 3638 Friedel pairs
Flack parameter: −0.03 (10)

### 

Data collection: *CrystalClear* (Rigaku/MSC 2005 ▶); cell refinement: *CrystalClear*; data reduction: *CrystalClear*; program(s) used to solve structure: *SHELXS97* (Sheldrick, 2008 ▶); program(s) used to refine structure: *SHELXL97* (Sheldrick, 2008 ▶); molecular graphics: *DIAMOND* (Brandenburg, 2006 ▶); software used to prepare material for publication: *SHELXL97*.

## Supplementary Material

Crystal structure: contains datablocks global, I. DOI: 10.1107/S1600536809047072/hb5216sup1.cif

Structure factors: contains datablocks I. DOI: 10.1107/S1600536809047072/hb5216Isup2.hkl

Additional supplementary materials:  crystallographic information; 3D view; checkCIF report

## Figures and Tables

**Table 1:** Hydrogen-bond geometry (Å, °)

*D*—H⋯*A*	*D*—H	H⋯*A*	*D*⋯*A*	*D*—H⋯*A*
N1—H1n⋯O2^i^	0.88	1.87	2.749 (3)	177
N2—H2n⋯O3^i^	0.88	1.95	2.806 (3)	165
N5—H5a⋯N4	0.92	2.16	2.912 (3)	138
N5—H5a⋯O3^i^	0.92	2.40	3.111 (3)	134
N5—H5b⋯O4^ii^	0.92	2.03	2.858 (3)	149
O4—H4o⋯N3^iii^	0.84	1.98	2.805 (4)	168

Symmetry codes: (i) 
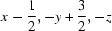
; (ii) 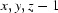; (iii) 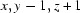.

**Fig. 1.**
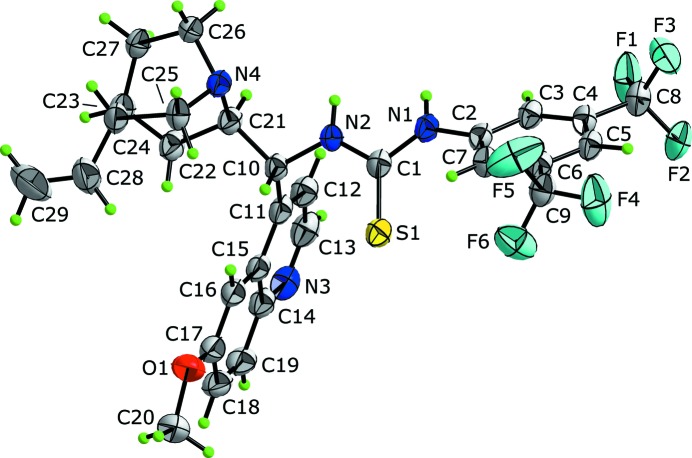
Molecular structure of the disubstituted urea molecule in (I), showing displacement ellipsoids at the 70% probability level.

**Fig. 2.**
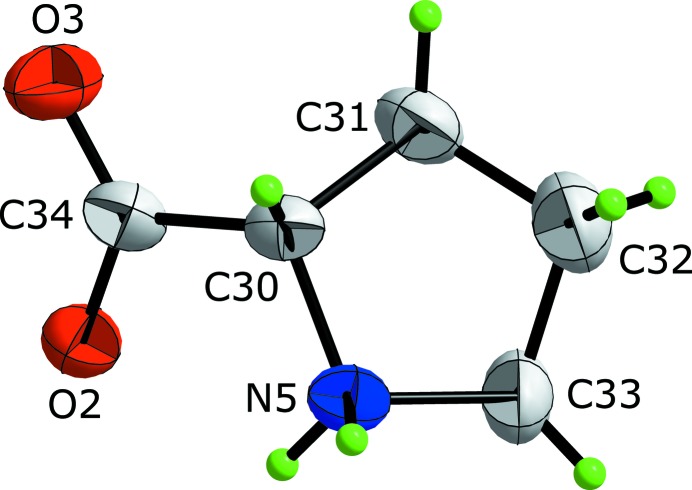
Molecular structure of zwitterionic L-proline, showing displacement ellipsoids at the 70% probability level.

**Fig. 3.**
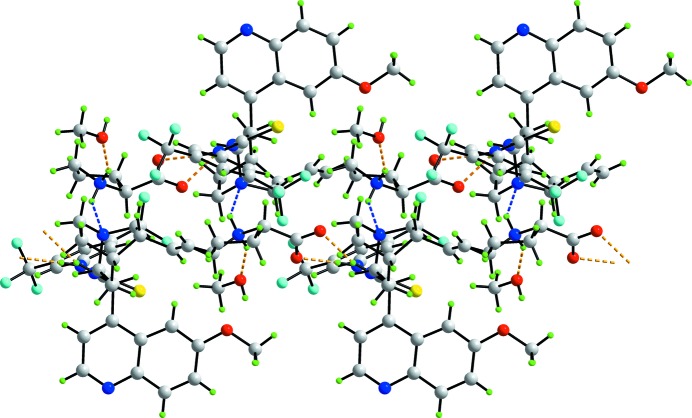
Supramolecular chain along the a axis in (I) mediated by N–H···O (orange dashed lines) and N–H···N hydrogen bonds (blue dashed lines).

**Fig. 4.**
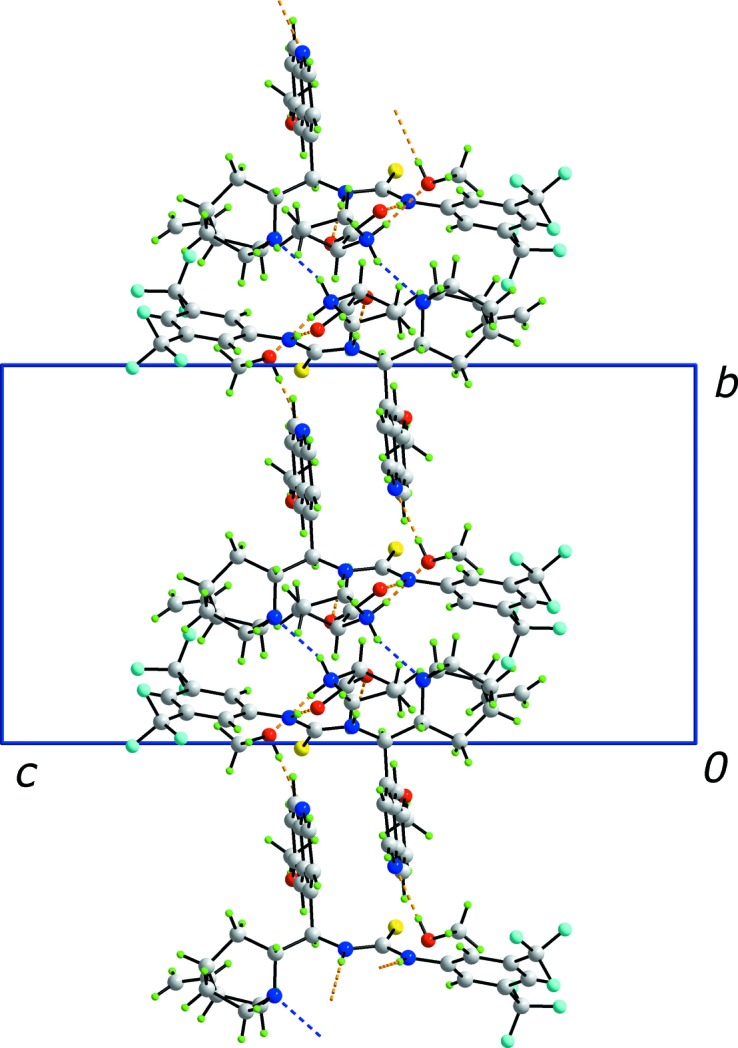
2-D array in the ab plane in (I) mediated by N–H···O (orange dashed lines) and N–H···N hydrogen bonds (blue dashed lines).

## supplementary crystallographic information

### Comment

The title co-crystal (Zukerman-Schpector & Tiekink, 2008), (I), has been evaluated as a pre-catalyst for the Michael addition of acetone to *trans*-β-nitrostyrene (Mandal & Zhao, 2008; Muramulla *et al.*, 2009). The combination of quinidine thiourea and L-proline is activating both the nucleophile and electrophile of the Michael reactions. The asymmetric Michael addition of acidic carbon pronucleophiles to nitroolefins is an important carbon-carbon bond forming reaction that provides access to synthetically useful enantioenriched nitroalkanes.

The absolute structure of the co-crystal, isolated as a methanol solvate, (I), has been determined, Figs 1 and 2, and reveals the chirality at the N4, C10, C21, C23, C24 and C30 atoms of the disubstituted thiourea molecule to be S, *R*, *R*, *R*, *S* and *S*, respectively. The L-proline molecule exists as a zwitterion, a conclusion confirmed by the equality of the C35–O2 (1.257 (3) Å) and C35–O3 distances (1.250 (4) Å), and by the pattern of hydrogen bonding interactions involving both ammonium-H atoms. The proline ring conformation is an envelope on atom C(32).

In the crystal structure, molecules are connected into a supramolecular chain along the *a* axis which, in turn, are connected into layers in the *ab* plane, Table 1. Each N–H atom of the disubstituted urea molecule is hydrogen bonded to a carboxylate-O atom. One of the ammonium-H atoms of the proline molecule links a neighbouring molecule by forming an N5–H5a···N4 hydrogen bond with the nitrogen atom of the dabco residue; the H5a atom also forms a weak N–H···O contact with a carboxylate-O3 atom to provide extra stability to the chain. The second ammonium-H forms a N–H···O hydrogen bond with the solvent methanol molecule. As shown in Fig. 3, the hydrogen bonding scheme described thus far leads to the formation of a supramolecular chain. The pyridine-N3 atoms are directed to the periphery of this chain and these hydrogen bond with the methanol molecule to form links between chains to generate a 2-D array, Fig. 4.

### Experimental

Compound (I) was prepared from the reaction of quinidine thiourea (30 mg, 0.05 mmol), prepared using a literature procedure (Vakulya *et al.*, 2005), and L-proline (Sigma Aldrich; 0.05 mmol) in a 1:1 ratio in methanol (2 ml). The vial was left uncorked and kept in a beaker half filled with pentane sealed with parafilm. After 1 day, crystals were isolated.

### Refinement

The H atoms were geometrically placed (O—H = 0.84 Å and C—H = 0.95–1.00 Å) and refined as riding with *U*_iso_(H) = 1.2*U*_eq_(C) or 1.5*U*_eq_(O, methyl-C). In the absence of significant anomalous scattering effects, 1951 Friedel pairs were averaged in the final refinement. The absolute configuration was determined on the basis of the known configuration of L-proline starting material.

### Crystal data

**Table:**

C_29_H_28_F_6_N_4_OS·C_5_H_9_NO_2_·CH_4_O	*F*(000) = 1552
*M**_r_* = 741.79	*D*_x_ = 1.362 Mg m^−^^3^
Orthorhombic, *P*2_1_2_1_2_1_	Mo *K*α radiation, λ = 0.71073 Å
Hall symbol: P 2ac 2ab	Cell parameters from 16196 reflections
*a* = 11.597 (3) Å	θ = 2.3–40.2°
*b* = 13.044 (4) Å	µ = 0.17 mm^−^^1^
*c* = 23.907 (7) Å	*T* = 98 K
*V* = 3616.4 (18) Å^3^	Plate, colourless
*Z* = 4	0.28 × 0.25 × 0.05 mm

### Data collection

**Table:**

Rigaku AFC12K/SATURN724 diffractometer	8250 independent reflections
Radiation source: fine-focus sealed tube	7519 reflections with *I* > 2σ(*I*)
graphite	*R*_int_ = 0.059
ω scans	θ_max_ = 27.5°, θ_min_ = 2.3°
Absorption correction: multi-scan (*ABSCOR*; Higashi, 1995)	*h* = −12→15
*T*_min_ = 0.722, *T*_max_ = 1.000	*k* = −16→16
26093 measured reflections	*l* = −31→30

### Refinement

**Table:**

Refinement on *F*^2^	Secondary atom site location: difference Fourier map
Least-squares matrix: full	Hydrogen site location: inferred from neighbouring sites
*R*[*F*^2^ > 2σ(*F*^2^)] = 0.060	H-atom parameters constrained
*wR*(*F*^2^) = 0.149	*w* = 1/[σ^2^(*F*_o_^2^) + (0.0632*P*)^2^ + 2.0147*P*] where *P* = (*F*_o_^2^ + 2*F*_c_^2^)/3
*S* = 1.06	(Δ/σ)_max_ < 0.001
8250 reflections	Δρ_max_ = 0.44 e Å^−^^3^
471 parameters	Δρ_min_ = −0.27 e Å^−^^3^
3 restraints	Absolute structure: Flack (1983), 3638 Friedel pairs
Primary atom site location: structure-invariant direct methods	Flack parameter: −0.03 (10)

### Special details

**Table:**

Geometry. All s.u.'s (except the s.u. in the dihedral angle between two l.s. planes) are estimated using the full covariance matrix. The cell s.u.'s are taken into account individually in the estimation of s.u.'s in distances, angles and torsion angles; correlations between s.u.'s in cell parameters are only used when they are defined by crystal symmetry. An approximate (isotropic) treatment of cell s.u.'s is used for estimating s.u.'s involving l.s. planes.
Refinement. Refinement of *F*^2^ against ALL reflections. The weighted *R*-factor *wR* and goodness of fit *S* are based on *F*^2^, conventional *R*-factors *R* are based on *F*, with *F* set to zero for negative *F*^2^. The threshold expression of *F*^2^ > 2σ(*F*^2^) is used only for calculating *R*-factors(gt) *etc*. and is not relevant to the choice of reflections for refinement. *R*-factors based on *F*^2^ are statistically about twice as large as those based on *F*, and *R*- factors based on ALL data will be even larger.

### Fractional atomic coordinates and isotropic or equivalent isotropic displacement parameters (Å^2^)

**Table:**

	*x*	*y*	*z*	*U*_iso_*/*U*_eq_	
S1	0.20467 (6)	1.01378 (6)	0.06774 (3)	0.03013 (16)	
F1	−0.27893 (18)	0.9872 (2)	0.24180 (9)	0.0572 (6)	
F2	−0.16591 (19)	1.00733 (17)	0.31179 (9)	0.0499 (5)	
F3	−0.23783 (18)	0.86002 (16)	0.29538 (10)	0.0489 (5)	
F4	0.2087 (2)	0.8036 (2)	0.30422 (9)	0.0617 (7)	
F5	0.2224 (2)	0.71239 (17)	0.22931 (13)	0.0701 (8)	
F6	0.30477 (17)	0.85610 (18)	0.23501 (10)	0.0513 (6)	
O1	0.52077 (19)	1.13957 (19)	−0.08112 (11)	0.0406 (6)	
N1	−0.0075 (2)	0.9329 (2)	0.08569 (10)	0.0266 (5)	
H1N	−0.0783	0.9226	0.0737	0.032*	
N2	0.0423 (2)	0.95532 (18)	−0.00465 (10)	0.0244 (5)	
H2N	−0.0232	0.9231	−0.0106	0.029*	
N3	0.0920 (3)	1.3256 (2)	−0.06678 (12)	0.0354 (6)	
N4	0.0730 (2)	0.83257 (18)	−0.10719 (10)	0.0251 (5)	
C1	0.0756 (2)	0.9653 (2)	0.04910 (12)	0.0252 (5)	
C2	0.0023 (3)	0.9169 (2)	0.14352 (12)	0.0262 (6)	
C3	−0.0951 (3)	0.9368 (2)	0.17688 (12)	0.0264 (6)	
H3	−0.1636	0.9628	0.1604	0.032*	
C4	−0.0906 (3)	0.9185 (2)	0.23392 (12)	0.0277 (6)	
C5	0.0068 (3)	0.8775 (2)	0.25935 (12)	0.0274 (6)	
H5	0.0087	0.8649	0.2985	0.033*	
C6	0.1016 (3)	0.8556 (2)	0.22551 (12)	0.0267 (6)	
C7	0.1005 (3)	0.8754 (2)	0.16783 (12)	0.0273 (6)	
H7	0.1663	0.8606	0.1456	0.033*	
C8	−0.1931 (3)	0.9433 (2)	0.27009 (13)	0.0326 (6)	
C9	0.2074 (3)	0.8054 (2)	0.24908 (12)	0.0314 (6)	
C10	0.1097 (2)	0.9926 (2)	−0.05211 (11)	0.0240 (5)	
H10	0.1914	0.9693	−0.0481	0.029*	
C11	0.1065 (3)	1.1100 (2)	−0.05523 (11)	0.0268 (6)	
C12	0.0038 (3)	1.1606 (2)	−0.05184 (13)	0.0317 (6)	
H12	−0.0652	1.1233	−0.0451	0.038*	
C13	−0.0004 (3)	1.2689 (2)	−0.05832 (14)	0.0358 (7)	
H13	−0.0732	1.3020	−0.0564	0.043*	
C14	0.1968 (3)	1.2774 (2)	−0.06935 (12)	0.0303 (6)	
C15	0.2093 (3)	1.1689 (2)	−0.06446 (12)	0.0272 (5)	
C16	0.3214 (3)	1.1273 (2)	−0.06837 (13)	0.0291 (6)	
H16	0.3315	1.0553	−0.0650	0.035*	
C17	0.4170 (3)	1.1892 (2)	−0.07707 (13)	0.0331 (7)	
C18	0.4039 (3)	1.2961 (2)	−0.08108 (13)	0.0367 (7)	
H18	0.4696	1.3385	−0.0863	0.044*	
C19	0.2961 (3)	1.3391 (2)	−0.07744 (13)	0.0353 (7)	
H19	0.2879	1.4114	−0.0804	0.042*	
C20	0.6235 (3)	1.2008 (3)	−0.08248 (16)	0.0441 (9)	
H20A	0.6225	1.2446	−0.1158	0.066*	
H20B	0.6912	1.1560	−0.0837	0.066*	
H20C	0.6269	1.2438	−0.0489	0.066*	
C21	0.0576 (3)	0.9460 (2)	−0.10557 (11)	0.0253 (5)	
H21	−0.0272	0.9595	−0.1044	0.030*	
C22	0.1037 (3)	0.9946 (2)	−0.16008 (12)	0.0316 (6)	
H22A	0.0518	1.0504	−0.1725	0.038*	
H22B	0.1814	1.0238	−0.1538	0.038*	
C23	0.1093 (3)	0.9104 (2)	−0.20482 (12)	0.0296 (6)	
H23	0.1227	0.9413	−0.2426	0.035*	
C24	0.2072 (3)	0.8353 (2)	−0.19002 (12)	0.0309 (6)	
H24	0.1966	0.7716	−0.2127	0.037*	
C25	0.1918 (3)	0.8074 (2)	−0.12748 (12)	0.0287 (6)	
H25A	0.2063	0.7332	−0.1223	0.034*	
H25B	0.2491	0.8454	−0.1049	0.034*	
C26	−0.0115 (3)	0.7913 (2)	−0.14802 (12)	0.0286 (6)	
H26A	−0.0904	0.7976	−0.1325	0.034*	
H26B	0.0042	0.7177	−0.1545	0.034*	
C27	−0.0043 (3)	0.8501 (2)	−0.20430 (12)	0.0298 (6)	
H27A	−0.0064	0.8013	−0.2360	0.036*	
H27B	−0.0705	0.8976	−0.2080	0.036*	
C28	0.3260 (3)	0.8790 (4)	−0.20272 (16)	0.0503 (10)	
H28	0.3631	0.9173	−0.1741	0.060*	
C29	0.3799 (4)	0.8677 (3)	−0.2495 (2)	0.0744 (16)	
H29A	0.3455	0.8298	−0.2791	0.089*	
H29B	0.4541	0.8972	−0.2544	0.089*	
O2	0.27276 (18)	0.59342 (16)	−0.04507 (9)	0.0308 (5)	
O3	0.3580 (2)	0.67889 (19)	0.02523 (10)	0.0397 (6)	
N5	0.0627 (2)	0.66700 (18)	−0.02563 (10)	0.0251 (5)	
H5A	0.0321	0.7254	−0.0412	0.030*	
H5B	0.0929	0.6274	−0.0539	0.030*	
C30	0.1558 (2)	0.6948 (2)	0.01543 (12)	0.0254 (6)	
H30	0.1605	0.7710	0.0195	0.030*	
C31	0.1171 (3)	0.6463 (3)	0.07085 (13)	0.0367 (7)	
H31A	0.1510	0.5772	0.0756	0.044*	
H31B	0.1396	0.6896	0.1030	0.044*	
C32	−0.0125 (3)	0.6405 (3)	0.06533 (16)	0.0436 (8)	
H32A	−0.0486	0.7078	0.0728	0.052*	
H32B	−0.0452	0.5889	0.0913	0.052*	
C33	−0.0294 (3)	0.6086 (3)	0.00486 (15)	0.0400 (8)	
H33A	−0.0190	0.5337	0.0003	0.048*	
H33B	−0.1071	0.6279	−0.0087	0.048*	
C34	0.2729 (3)	0.6519 (2)	−0.00328 (12)	0.0274 (6)	
O4	0.0694 (2)	0.52089 (17)	0.88533 (10)	0.0431 (6)	
H4O	0.0870	0.4641	0.8995	0.065*	
C35	−0.0103 (3)	0.4973 (3)	0.84173 (15)	0.0406 (7)	
H36A	−0.0892	0.5029	0.8561	0.061*	
H36B	0.0034	0.4273	0.8284	0.061*	
H36C	0.0002	0.5457	0.8108	0.061*	

### Atomic displacement parameters (Å^2^)

**Table:**

	*U*^11^	*U*^22^	*U*^33^	*U*^12^	*U*^13^	*U*^23^
S1	0.0295 (3)	0.0384 (4)	0.0225 (3)	−0.0023 (3)	−0.0030 (3)	0.0013 (3)
F1	0.0466 (11)	0.0944 (18)	0.0307 (10)	0.0325 (13)	0.0052 (9)	0.0051 (11)
F2	0.0609 (13)	0.0517 (12)	0.0370 (11)	0.0014 (10)	0.0119 (10)	−0.0207 (10)
F3	0.0517 (12)	0.0437 (11)	0.0512 (13)	−0.0029 (9)	0.0233 (11)	0.0025 (10)
F4	0.0498 (12)	0.108 (2)	0.0278 (10)	0.0225 (14)	−0.0036 (10)	0.0171 (12)
F5	0.0691 (16)	0.0384 (12)	0.103 (2)	0.0207 (11)	−0.0424 (16)	−0.0196 (13)
F6	0.0359 (10)	0.0605 (14)	0.0576 (14)	−0.0024 (10)	−0.0059 (10)	0.0194 (11)
O1	0.0327 (11)	0.0404 (13)	0.0486 (15)	−0.0101 (10)	0.0050 (11)	−0.0062 (11)
N1	0.0281 (11)	0.0337 (13)	0.0180 (11)	−0.0001 (10)	−0.0024 (10)	0.0006 (9)
N2	0.0277 (11)	0.0269 (12)	0.0185 (11)	−0.0027 (9)	−0.0002 (9)	0.0019 (9)
N3	0.0502 (16)	0.0284 (12)	0.0277 (13)	0.0041 (11)	−0.0036 (13)	−0.0010 (11)
N4	0.0308 (12)	0.0245 (11)	0.0199 (11)	−0.0001 (9)	−0.0005 (10)	−0.0007 (9)
C1	0.0322 (14)	0.0230 (13)	0.0206 (12)	0.0027 (11)	0.0011 (11)	0.0000 (10)
C2	0.0342 (14)	0.0238 (13)	0.0205 (13)	−0.0004 (11)	0.0010 (12)	−0.0031 (10)
C3	0.0312 (14)	0.0264 (13)	0.0215 (13)	0.0022 (11)	−0.0018 (11)	0.0007 (10)
C4	0.0336 (14)	0.0270 (13)	0.0226 (13)	0.0027 (12)	0.0029 (12)	−0.0025 (11)
C5	0.0360 (15)	0.0276 (13)	0.0188 (13)	0.0021 (12)	0.0002 (12)	0.0006 (10)
C6	0.0327 (14)	0.0271 (14)	0.0204 (13)	0.0004 (12)	−0.0019 (12)	0.0006 (10)
C7	0.0295 (14)	0.0299 (14)	0.0227 (13)	0.0036 (12)	0.0024 (11)	−0.0012 (11)
C8	0.0396 (16)	0.0355 (15)	0.0228 (14)	0.0050 (13)	0.0025 (13)	−0.0006 (12)
C9	0.0345 (15)	0.0348 (15)	0.0250 (14)	0.0054 (13)	−0.0028 (13)	0.0015 (11)
C10	0.0298 (13)	0.0240 (13)	0.0181 (12)	−0.0004 (11)	0.0001 (10)	0.0020 (10)
C11	0.0381 (15)	0.0252 (13)	0.0171 (13)	0.0017 (11)	−0.0005 (12)	−0.0009 (10)
C12	0.0375 (15)	0.0311 (15)	0.0266 (14)	0.0014 (13)	−0.0012 (13)	0.0003 (11)
C13	0.0458 (18)	0.0315 (15)	0.0302 (16)	0.0088 (14)	−0.0041 (15)	−0.0028 (12)
C14	0.0432 (16)	0.0253 (13)	0.0224 (13)	−0.0021 (12)	−0.0013 (13)	0.0007 (11)
C15	0.0395 (15)	0.0251 (13)	0.0171 (12)	−0.0025 (12)	−0.0018 (12)	−0.0010 (10)
C16	0.0378 (15)	0.0274 (13)	0.0220 (13)	−0.0024 (11)	−0.0002 (12)	−0.0027 (11)
C17	0.0418 (17)	0.0343 (16)	0.0233 (15)	−0.0095 (13)	−0.0005 (13)	−0.0033 (11)
C18	0.0502 (19)	0.0310 (16)	0.0291 (16)	−0.0145 (14)	−0.0032 (14)	−0.0004 (12)
C19	0.0545 (18)	0.0221 (13)	0.0293 (16)	−0.0095 (14)	0.0004 (15)	0.0007 (11)
C20	0.0378 (17)	0.052 (2)	0.043 (2)	−0.0150 (16)	0.0059 (15)	−0.0086 (16)
C21	0.0319 (14)	0.0237 (13)	0.0202 (12)	−0.0012 (11)	−0.0023 (11)	0.0025 (10)
C22	0.0472 (17)	0.0266 (14)	0.0209 (13)	−0.0050 (13)	−0.0010 (12)	0.0037 (11)
C23	0.0342 (15)	0.0331 (15)	0.0214 (13)	−0.0057 (12)	−0.0027 (12)	0.0029 (11)
C24	0.0295 (14)	0.0423 (16)	0.0210 (13)	0.0002 (13)	0.0020 (12)	−0.0027 (12)
C25	0.0321 (15)	0.0326 (15)	0.0213 (13)	0.0047 (12)	0.0009 (11)	0.0017 (11)
C26	0.0330 (15)	0.0309 (14)	0.0217 (13)	−0.0074 (12)	0.0004 (12)	−0.0019 (11)
C27	0.0344 (14)	0.0343 (15)	0.0207 (13)	−0.0027 (12)	−0.0036 (12)	0.0001 (11)
C28	0.0372 (18)	0.087 (3)	0.0270 (17)	−0.0125 (18)	0.0021 (14)	0.0054 (18)
C29	0.073 (3)	0.051 (2)	0.098 (4)	−0.024 (2)	0.049 (3)	−0.013 (3)
O2	0.0304 (11)	0.0346 (11)	0.0275 (10)	−0.0001 (9)	0.0004 (9)	−0.0076 (8)
O3	0.0342 (12)	0.0472 (14)	0.0376 (13)	0.0069 (10)	−0.0082 (10)	−0.0150 (11)
N5	0.0296 (12)	0.0230 (11)	0.0227 (11)	0.0038 (9)	−0.0034 (10)	−0.0015 (9)
C30	0.0287 (14)	0.0256 (13)	0.0220 (14)	−0.0002 (11)	−0.0039 (11)	−0.0001 (10)
C31	0.0424 (17)	0.0456 (18)	0.0221 (14)	0.0013 (14)	0.0009 (14)	0.0018 (13)
C32	0.0428 (18)	0.0484 (19)	0.0396 (19)	−0.0055 (15)	0.0124 (17)	−0.0022 (16)
C33	0.0317 (16)	0.0484 (19)	0.0400 (19)	−0.0103 (14)	0.0109 (14)	−0.0094 (15)
C34	0.0336 (15)	0.0263 (14)	0.0222 (13)	0.0029 (11)	−0.0019 (12)	0.0001 (10)
O4	0.0703 (16)	0.0265 (11)	0.0324 (12)	0.0038 (11)	−0.0218 (12)	−0.0040 (9)
C35	0.0451 (18)	0.0412 (18)	0.0354 (17)	0.0002 (15)	−0.0088 (15)	−0.0022 (14)

### Geometric parameters (Å, °)

**Table:**

S1—C1	1.685 (3)	C20—H20A	0.9800
F1—C8	1.333 (4)	C20—H20B	0.9800
F2—C8	1.338 (4)	C20—H20C	0.9800
F3—C8	1.348 (4)	C21—C22	1.545 (4)
F4—C9	1.319 (4)	C21—H21	1.0000
F5—C9	1.314 (4)	C22—C23	1.534 (4)
F6—C9	1.351 (4)	C22—H22A	0.9900
O1—C17	1.370 (4)	C22—H22B	0.9900
O1—C20	1.435 (4)	C23—C27	1.535 (4)
N1—C1	1.369 (4)	C23—C24	1.541 (4)
N1—C2	1.403 (4)	C23—H23	1.0000
N1—H1N	0.8799	C24—C28	1.521 (4)
N2—C1	1.348 (4)	C24—C25	1.549 (4)
N2—C10	1.461 (3)	C24—H24	1.0000
N2—H2N	0.8799	C25—H25A	0.9900
N3—C13	1.317 (4)	C25—H25B	0.9900
N3—C14	1.370 (4)	C26—C27	1.551 (4)
N4—C26	1.484 (4)	C26—H26A	0.9900
N4—C21	1.491 (4)	C26—H26B	0.9900
N4—C25	1.497 (4)	C27—H27A	0.9900
C2—C7	1.389 (4)	C27—H27B	0.9900
C2—C3	1.406 (4)	C28—C29	1.290 (6)
C3—C4	1.385 (4)	C28—H28	0.9500
C3—H3	0.9500	C29—H29A	0.9500
C4—C5	1.389 (4)	C29—H29B	0.9500
C4—C8	1.505 (4)	O2—C34	1.257 (3)
C5—C6	1.395 (4)	O3—C34	1.250 (4)
C5—H5	0.9500	N5—C33	1.501 (4)
C6—C7	1.403 (4)	N5—C30	1.504 (4)
C6—C9	1.500 (4)	N5—H5A	0.9200
C7—H7	0.9500	N5—H5B	0.9200
C10—C11	1.533 (4)	C30—C34	1.535 (4)
C10—C21	1.538 (4)	C30—C31	1.535 (4)
C10—H10	1.0000	C30—H30	1.0000
C11—C12	1.364 (4)	C31—C32	1.511 (5)
C11—C15	1.436 (4)	C31—H31A	0.9900
C12—C13	1.423 (4)	C31—H31B	0.9900
C12—H12	0.9500	C32—C33	1.517 (5)
C13—H13	0.9500	C32—H32A	0.9900
C14—C19	1.418 (4)	C32—H32B	0.9900
C14—C15	1.427 (4)	C33—H33A	0.9900
C15—C16	1.411 (4)	C33—H33B	0.9900
C16—C17	1.387 (4)	O4—C35	1.426 (4)
C16—H16	0.9500	O4—H4O	0.8400
C17—C18	1.406 (5)	C35—H36A	0.9800
C18—C19	1.373 (5)	C35—H36B	0.9800
C18—H18	0.9500	C35—H36C	0.9800
C19—H19	0.9500		
			
C17—O1—C20	117.9 (3)	N4—C21—H21	107.0
C1—N1—C2	128.2 (3)	C10—C21—H21	107.0
C1—N1—H1N	119.7	C22—C21—H21	107.0
C2—N1—H1N	112.1	C23—C22—C21	108.0 (2)
C1—N2—C10	123.7 (2)	C23—C22—H22A	110.1
C1—N2—H2N	116.5	C21—C22—H22A	110.1
C10—N2—H2N	119.7	C23—C22—H22B	110.1
C13—N3—C14	118.1 (3)	C21—C22—H22B	110.1
C26—N4—C21	107.4 (2)	H22A—C22—H22B	108.4
C26—N4—C25	108.4 (2)	C22—C23—C27	109.0 (2)
C21—N4—C25	109.7 (2)	C22—C23—C24	109.0 (2)
N2—C1—N1	112.2 (2)	C27—C23—C24	107.7 (2)
N2—C1—S1	122.9 (2)	C22—C23—H23	110.3
N1—C1—S1	124.9 (2)	C27—C23—H23	110.3
C7—C2—N1	122.5 (3)	C24—C23—H23	110.3
C7—C2—C3	119.5 (3)	C28—C24—C23	112.6 (3)
N1—C2—C3	117.8 (3)	C28—C24—C25	112.7 (3)
C4—C3—C2	119.8 (3)	C23—C24—C25	106.7 (2)
C4—C3—H3	120.1	C28—C24—H24	108.3
C2—C3—H3	120.1	C23—C24—H24	108.3
C3—C4—C5	121.8 (3)	C25—C24—H24	108.3
C3—C4—C8	119.9 (3)	N4—C25—C24	111.5 (2)
C5—C4—C8	118.2 (3)	N4—C25—H25A	109.3
C4—C5—C6	117.8 (3)	C24—C25—H25A	109.3
C4—C5—H5	121.1	N4—C25—H25B	109.3
C6—C5—H5	121.1	C24—C25—H25B	109.3
C5—C6—C7	121.6 (3)	H25A—C25—H25B	108.0
C5—C6—C9	121.1 (3)	N4—C26—C27	110.8 (2)
C7—C6—C9	117.2 (3)	N4—C26—H26A	109.5
C2—C7—C6	119.4 (3)	C27—C26—H26A	109.5
C2—C7—H7	120.3	N4—C26—H26B	109.5
C6—C7—H7	120.3	C27—C26—H26B	109.5
F1—C8—F2	106.6 (3)	H26A—C26—H26B	108.1
F1—C8—F3	106.6 (3)	C23—C27—C26	107.9 (2)
F2—C8—F3	105.1 (3)	C23—C27—H27A	110.1
F1—C8—C4	113.0 (3)	C26—C27—H27A	110.1
F2—C8—C4	112.1 (3)	C23—C27—H27B	110.1
F3—C8—C4	112.8 (3)	C26—C27—H27B	110.1
F5—C9—F4	110.0 (3)	H27A—C27—H27B	108.4
F5—C9—F6	104.6 (3)	C29—C28—C24	124.7 (4)
F4—C9—F6	104.4 (3)	C29—C28—H28	117.6
F5—C9—C6	112.1 (3)	C24—C28—H28	117.6
F4—C9—C6	113.1 (3)	C28—C29—H29A	120.0
F6—C9—C6	112.1 (2)	C28—C29—H29B	120.0
N2—C10—C11	111.0 (2)	H29A—C29—H29B	120.0
N2—C10—C21	107.7 (2)	C33—N5—C30	108.4 (2)
C11—C10—C21	110.2 (2)	C33—N5—H5A	110.0
N2—C10—H10	109.3	C30—N5—H5A	110.0
C11—C10—H10	109.3	C33—N5—H5B	110.0
C21—C10—H10	109.3	C30—N5—H5B	110.0
C12—C11—C15	118.4 (3)	H5A—N5—H5B	108.4
C12—C11—C10	120.1 (3)	N5—C30—C34	110.9 (2)
C15—C11—C10	121.5 (3)	N5—C30—C31	104.7 (2)
C11—C12—C13	120.2 (3)	C34—C30—C31	111.1 (2)
C11—C12—H12	119.9	N5—C30—H30	110.0
C13—C12—H12	119.9	C34—C30—H30	110.0
N3—C13—C12	123.1 (3)	C31—C30—H30	110.0
N3—C13—H13	118.4	C32—C31—C30	103.7 (3)
C12—C13—H13	118.4	C32—C31—H31A	111.0
N3—C14—C19	117.8 (3)	C30—C31—H31A	111.0
N3—C14—C15	122.8 (3)	C32—C31—H31B	111.0
C19—C14—C15	119.4 (3)	C30—C31—H31B	111.0
C16—C15—C14	118.0 (3)	H31A—C31—H31B	109.0
C16—C15—C11	124.7 (3)	C31—C32—C33	103.0 (3)
C14—C15—C11	117.3 (3)	C31—C32—H32A	111.2
C17—C16—C15	121.5 (3)	C33—C32—H32A	111.2
C17—C16—H16	119.3	C31—C32—H32B	111.2
C15—C16—H16	119.3	C33—C32—H32B	111.2
O1—C17—C16	116.0 (3)	H32A—C32—H32B	109.1
O1—C17—C18	123.9 (3)	N5—C33—C32	103.4 (3)
C16—C17—C18	120.1 (3)	N5—C33—H33A	111.1
C19—C18—C17	119.9 (3)	C32—C33—H33A	111.1
C19—C18—H18	120.0	N5—C33—H33B	111.1
C17—C18—H18	120.0	C32—C33—H33B	111.1
C18—C19—C14	121.1 (3)	H33A—C33—H33B	109.0
C18—C19—H19	119.5	O3—C34—O2	127.2 (3)
C14—C19—H19	119.5	O3—C34—C30	115.9 (2)
O1—C20—H20A	109.5	O2—C34—C30	116.9 (3)
O1—C20—H20B	109.5	C35—O4—H4O	105.2
H20A—C20—H20B	109.5	O4—C35—H36A	109.5
O1—C20—H20C	109.5	O4—C35—H36B	109.5
H20A—C20—H20C	109.5	H36A—C35—H36B	109.5
H20B—C20—H20C	109.5	O4—C35—H36C	109.5
N4—C21—C10	111.5 (2)	H36A—C35—H36C	109.5
N4—C21—C22	110.1 (2)	H36B—C35—H36C	109.5
C10—C21—C22	113.8 (2)		
			
C10—N2—C1—N1	174.2 (2)	C14—C15—C16—C17	−0.4 (4)
C10—N2—C1—S1	−5.1 (4)	C11—C15—C16—C17	−179.7 (3)
C2—N1—C1—N2	169.3 (3)	C20—O1—C17—C16	−171.7 (3)
C2—N1—C1—S1	−11.5 (4)	C20—O1—C17—C18	8.2 (5)
C1—N1—C2—C7	−38.0 (4)	C15—C16—C17—O1	−178.8 (3)
C1—N1—C2—C3	146.9 (3)	C15—C16—C17—C18	1.3 (5)
C7—C2—C3—C4	2.4 (4)	O1—C17—C18—C19	178.9 (3)
N1—C2—C3—C4	177.6 (3)	C16—C17—C18—C19	−1.2 (5)
C2—C3—C4—C5	−2.1 (4)	C17—C18—C19—C14	0.3 (5)
C2—C3—C4—C8	177.8 (3)	N3—C14—C19—C18	−179.3 (3)
C3—C4—C5—C6	0.4 (4)	C15—C14—C19—C18	0.5 (5)
C8—C4—C5—C6	−179.4 (3)	C26—N4—C21—C10	160.9 (2)
C4—C5—C6—C7	1.0 (4)	C25—N4—C21—C10	−81.6 (3)
C4—C5—C6—C9	−176.3 (3)	C26—N4—C21—C22	−71.8 (3)
N1—C2—C7—C6	−176.0 (3)	C25—N4—C21—C22	45.7 (3)
C3—C2—C7—C6	−1.0 (4)	N2—C10—C21—N4	−66.2 (3)
C5—C6—C7—C2	−0.7 (4)	C11—C10—C21—N4	172.7 (2)
C9—C6—C7—C2	176.7 (3)	N2—C10—C21—C22	168.5 (2)
C3—C4—C8—F1	−3.3 (4)	C11—C10—C21—C22	47.4 (3)
C5—C4—C8—F1	176.6 (3)	N4—C21—C22—C23	19.9 (3)
C3—C4—C8—F2	−123.8 (3)	C10—C21—C22—C23	145.9 (3)
C5—C4—C8—F2	56.1 (4)	C21—C22—C23—C27	46.9 (3)
C3—C4—C8—F3	117.8 (3)	C21—C22—C23—C24	−70.5 (3)
C5—C4—C8—F3	−62.3 (4)	C22—C23—C24—C28	−75.7 (3)
C5—C6—C9—F5	112.8 (3)	C27—C23—C24—C28	166.2 (3)
C7—C6—C9—F5	−64.5 (4)	C22—C23—C24—C25	48.3 (3)
C5—C6—C9—F4	−12.2 (4)	C27—C23—C24—C25	−69.8 (3)
C7—C6—C9—F4	170.4 (3)	C26—N4—C25—C24	48.2 (3)
C5—C6—C9—F6	−129.9 (3)	C21—N4—C25—C24	−68.7 (3)
C7—C6—C9—F6	52.7 (4)	C28—C24—C25—N4	141.8 (3)
C1—N2—C10—C11	−71.9 (3)	C23—C24—C25—N4	17.8 (3)
C1—N2—C10—C21	167.5 (3)	C21—N4—C26—C27	50.5 (3)
N2—C10—C11—C12	−48.9 (4)	C25—N4—C26—C27	−67.9 (3)
C21—C10—C11—C12	70.2 (3)	C22—C23—C27—C26	−66.9 (3)
N2—C10—C11—C15	134.2 (3)	C24—C23—C27—C26	51.2 (3)
C21—C10—C11—C15	−106.7 (3)	N4—C26—C27—C23	15.6 (3)
C15—C11—C12—C13	1.0 (4)	C23—C24—C28—C29	−90.9 (5)
C10—C11—C12—C13	−176.0 (3)	C25—C24—C28—C29	148.4 (5)
C14—N3—C13—C12	−0.1 (5)	C33—N5—C30—C34	122.3 (3)
C11—C12—C13—N3	−1.1 (5)	C33—N5—C30—C31	2.4 (3)
C13—N3—C14—C19	−178.9 (3)	N5—C30—C31—C32	−26.2 (3)
C13—N3—C14—C15	1.4 (5)	C34—C30—C31—C32	−146.0 (3)
N3—C14—C15—C16	179.3 (3)	C30—C31—C32—C33	40.0 (3)
C19—C14—C15—C16	−0.5 (4)	C30—N5—C33—C32	22.2 (3)
N3—C14—C15—C11	−1.4 (4)	C31—C32—C33—N5	−38.3 (4)
C19—C14—C15—C11	178.8 (3)	N5—C30—C34—O3	173.5 (3)
C12—C11—C15—C16	179.4 (3)	C31—C30—C34—O3	−70.4 (3)
C10—C11—C15—C16	−3.6 (4)	N5—C30—C34—O2	−6.9 (4)
C12—C11—C15—C14	0.2 (4)	C31—C30—C34—O2	109.1 (3)
C10—C11—C15—C14	177.1 (2)		

### Hydrogen-bond geometry (Å, °)

**Table:**

*D*—H···*A*	*D*—H	H···*A*	*D*···*A*	*D*—H···*A*
N1—H1n···O2^i^	0.88	1.87	2.749 (3)	177
N2—H2n···O3^i^	0.88	1.95	2.806 (3)	165
N5—H5a···N4	0.92	2.16	2.912 (3)	138
N5—H5a···O3^i^	0.92	2.40	3.111 (3)	134
N5—H5b···O4^ii^	0.92	2.03	2.858 (3)	149
O4—H4o···N3^iii^	0.84	1.98	2.805 (4)	168

Symmetry codes: (i) *x*−1/2, −*y*+3/2, −*z*; (ii) *x*, *y*, *z*−1; (iii) *x*, *y*−1, *z*+1.
